# A Practical Treatise on Venereal Diseases; or Critical and Experimental Researches on Inoculation Applied to the Study of Those Affections, with a Therapeutical Summary and Special Formulary

**Published:** 1843-06

**Authors:** 


Art. VI.—*A Practical Treatise on Venereal Diseases; or Critical and Experimental Researches on Inoculation applied to the Study of those Affections, with a Therapeutical Summary and Special Formulary.* By Ph. Ricord, M. D., Surgeon of the Venereal Hospital of Paris, Clinical Professor of Special Pathology, &c. Translated from the French by Henry Pilkington Drummond, M. D. Philadelphia, Lea and Blanchard: 1843. pp. 256.

Few members of the profession, who keep pace with its literature and its improvements, are unacquainted with the name of Ricord, in connexion with venereal diseases. By well directed study, and a series of happy experiments, he has done more perhaps than any living writer towards removing the obscurity that reigned over many important and interesting questions connected with those affections. As the result of his labors, we have a more rational pathology, a more exact diagnosis, and an improved system of therapeutics.

We have here his views presented in an English dress, which cannot fail to prove highly acceptable to the profession. Dr. Drummond has acquitted himself very well as a translator; for we have reason to know that the author's style presented great difficulties. Had he adhered less closely to the original, the translation would have been freer from certain gallicisms and redundancies; but it is clear—and that is sufficient. C.

